# Landscape Composition and Forest Structure Shape Phyllostomid Bat Assemblages in the Atlantic Forest Remnants

**DOI:** 10.3390/ani15142082

**Published:** 2025-07-15

**Authors:** Ricardo Bovendorp, Eduardo Mariano-Neto, Albérico Queiroz, Deborah Faria

**Affiliations:** 1 Applied Ecology and Conservation Laboratory, Departamento de Ciências Biológicas, Universidade Estadual de Santa Cruz, Ilhéus 45662-900, Bahia, Brazil; rsbovendorp@uesc.br (R.B.); marianon@gmail.com (E.M.-N.); alberico.queiroz@gmail.com (A.Q.); 2 Instituto de Biologia, Universidade Federal da Bahia, Salvador 40170-115, Bahia, Brazil

**Keywords:** richness, abundance, Chiroptera, forest cover, forest structure, pastures

## Abstract

Habitat loss and land-use intensification are major threats to biodiversity in the Brazilian Atlantic Forest, particularly for bat assemblages that provide key ecosystem services. In this study, we examined how landscape composition (forest and pasture cover) and local forest structure influence the richness and abundance of phyllostomid bats across 20 forest fragments in southern Bahia. Bat sampling was conducted using mist nets, and forest structure was quantified using tree measurements and vertical foliage stratification. We applied structural equation modeling to test the direct and indirect effects of landscape and local variables. Our results show that forest cover has both direct and indirect positive effects on bat diversity, mediated by improved forest structure. In contrast, increased pasture cover negatively affected forest structure and was weakly associated with bat diversity. The most abundant species were generalist frugivores, such as *Carollia perspicillata* and *Rhinophylla pumilio*. These findings highlight the importance of maintaining forest cover and structural complexity to support bat diversity in agroforestry-dominated landscapes. Conservation strategies that integrate habitat protection with sustainable land-use practices are crucial to maintaining biodiversity and the ecological functions provided by bats in this globally threatened biome.

## Simple Summary

Phyllostomid bats play vital roles in tropical ecosystems by pollinating plants, dispersing seeds, and controlling insects. However, their habitats in the Atlantic Forest of Brazil are increasingly threatened by deforestation and the spread of pastures. In this study, we explored how the amount of forest and pasture in the landscape, as well as the condition of local forest fragments, influences the number and abundance of these bats. We studied 20 forest fragments within cocoa-growing areas in southern Bahia and found that landscapes with more forest cover supported more bat species and higher numbers of individuals. This positive effect occurred both directly and by helping maintain healthy forest structure. In contrast, areas with more pasture had simpler forest structures and fewer bats. These results show that preserving forest areas and improving the quality of remaining forest fragments are essential for protecting bat populations and the services they provide. Our findings support the importance of land management practices that balance agriculture and conservation, offering practical ways to maintain biodiversity and ecosystem health in one of the world’s most endangered forests.

## 1. Introduction

Over the last century, anthropogenic disturbances such as deforestation, agricultural expansion, and pasture establishment have been recognized as major drivers of biodiversity loss, leading to habitat fragmentation and population declines across multiple taxa [1,2,3]. The conversion of natural habitats into agricultural systems has resulted in mosaic landscapes characterized by remnants of forests interspersed with anthropogenic habitats [4,5]. Within these fragmented systems, researchers have explored strategies to mitigate the negative effects of land-use change, with increasing attention to agroforestry systems (e.g., shade-grown cocoa, coffee, and rubber tree plantations) as potential biodiversity refuges that harbor diverse faunal and floral communities while maintaining ecosystem services [6,7].

The composition and configuration of the surrounding landscape matrix is critical in shaping biodiversity patterns within fragmented ecosystems. Forest cover at the landscape scale is a key predictor of habitat quality and connectivity, influencing local vegetation structure and the diversity of sensitive species such as bats, birds, and small mammals [5,8,9,10]. In highly fragmented landscapes, reduced forest cover leads to degraded vegetation structure, often favoring generalist bat species like *Carollia perspicillata*, while habitat specialists such as *Chrotopterus auritus* tend to decline [10,11,12,13]. Moreover, landscapes retaining less than 40% forest cover show marked reductions in ecological processes and species interactions, highlighting the need for targeted conservation efforts to maintain forest remnants [14,15].

Phyllostomid bats, one of the most ecologically diverse mammalian families, play critical roles in tropical ecosystems, including seed dispersal, pollination, and arthropod population regulation [16,17]. These ecological functions are essential for maintaining forest regeneration and ecosystem stability, as bats disperse seeds of over 500 plant species and contribute to pest control in agricultural systems [18,19,20]. Due to their wide range of habitat use and trophic diversity, phyllostomid bats are considered bioindicators of habitat quality and ecosystem integrity, with their populations responding to changes in forest structure and landscape composition [9,21,22].

The Atlantic Forest of Brazil, a global biodiversity hotspot, has experienced extensive deforestation, with around 23% of its original cover remaining as small, isolated fragments embedded in human-modified landscapes [23,24]. In southern Bahia, one of the biome’s centers of endemism, forest loss has significantly impacted ecological processes and biodiversity, including birds, mammals, and bats [9,11,12,25]. Agroforestry systems, particularly shade-grown cocoa, have been identified as valuable landscapes for conserving biodiversity in this region, although their effectiveness depends on the surrounding landscape matrix and forest cover [15,26,27].

Bats exhibit contrasting responses to habitat loss and landscape composition, influenced by factors such as species guilds and mobility. For example, frugivorous bats are often resilient to habitat loss due to their ability to exploit resources across fragmented landscapes [28,29]. In contrast, nectarivorous and carnivorous bats are more sensitive to forest loss, showing declines in abundance and activity [28,30]. Recent research in southern Bahia indicates that insectivorous bats are more affected by the configuration and composition of the landscape, highlighting the importance of understanding species-specific responses to anthropogenic disturbances [31]. However, similar studies focusing on phyllostomid bats remain scarce, despite their critical ecological roles.

To address these gaps, we used structural equation modeling (SEM) to investigate the direct and indirect effects of landscape factors (forest cover and pasture) and local factors (forest structure) on the diversity of phyllostomid bats across 20 forest fragments in the Atlantic Forest of southern Bahia, Brazil. Our conceptual model proposed that forest cover positively and directly influences forest structure and bat diversity, while also exerting an indirect effect on bat diversity via forest structure [9,32]. Conversely, pasture was hypothesized to negatively affect forest structure and bat diversity, both directly and indirectly highlighting habitat simplification [33,34]. By integrating landscape and local factors, this study aimed to elucidate the mechanisms driving phyllostomid bat diversity in fragmented tropical landscapes, with implications for conservation strategies in the Atlantic Forest.

## 2. Materials and Methods

### 2.1. The Study Area

**Figure 1:**
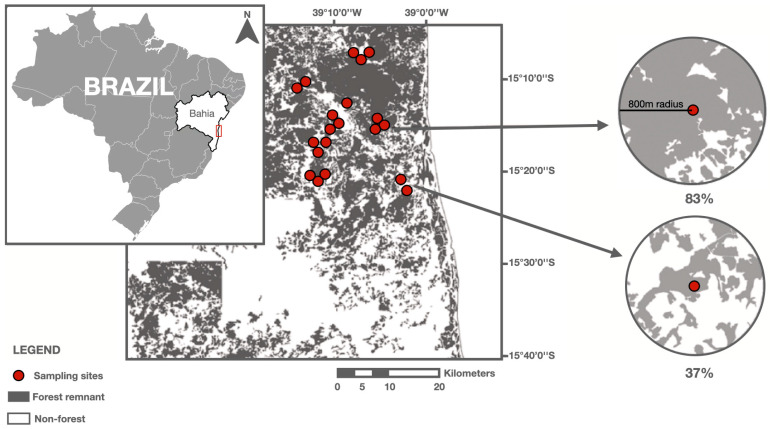
Distribution of the 20 forest fragments located in Atlantic Forest remnants in southern Bahia, Brazil. Two example landscapes (800 m radius) are also presented to illustrate differences in forest cover and configuration.

The study area is comprised by two sub-regions in the Atlantic Forest domain in the state of Bahia, northeastern Brazil. The 20 forest fragments are located predominantly in the municipalities of Una and Santa Luzia, separated by approximately 40 km (Figure 1).

The vegetation of both areas is classified as Dense Ombrophilous Forest [35], with canopy layers (25–30 m) and emergent trees (up to 40 m) with abundant epiphytes, ferns, bromeliads, and lianas [36]. Temperature ranges averaged 24 °C and rainfall 1500 mm year, with no clear seasonality [37].

To categorize the different vegetational formations present in the study area, we used high-resolution satellite images (RapidEye, Berlin, Germany, from 2009 to 2010, QuickBird 2, Colorado, USA and WordView, Vandemberg, USA from 2011) [23]. Both regions are characterized by agricultural land uses such as pastures; shade cacao agroforests; and eucalyptus and rubber tree plantations. In Santa Luzia, approximately 34% of the land is occupied by native vegetation, with pastures as the dominant land use occupying 60% of the region. Una region has the higher amount of forest cover, including shade cacao agroforests and rubber tree plantations representing approximately 54% of its area. We selected 20 forest fragments, each containing one sampling site, totaling 20 study sites used in this study [23]. Sites represented a gradient of forest cover ranging from 5.4 to 85.1% and were located at least 1 km from each other.

### 2.2. Landscape Variables

We quantified the amount of forest cover around the sampling site within multiple spatial scales starting from 100 m up to 2000 m radius using QGis version 3.4 [38]. We decide to not use larger scales because it would imply a significant increase in the overlapping of buffers, which leads to non-independence between the observations, compromising our analyses [39]. For each sampling site, we calculated the amount of forest cover and the proportion of matrix occupied by pastures. Forest cover was calculated as the total amount of forest (initial, intermediate, and advanced) divided by the buffer size. The proportion of pastures in landscape was calculated by pasture area/(buffer area—forest area). We used this metric to quantify matrix composition because pastures are a dominant land use in the area (40.4%) and the main land use with a non-forest structure compared to shade cocoa, eucalypt, and rubber tree plantations. Additionally, we test the appropriate spatial scale with a ‘multifit’ package in R, version 4.4 by AIC criteria [40]. The best spatial scale was 800 m radius.

### 2.3. Forest Structure

Forest structure data were obtained from the SISBIOTA project, which established 260 vegetation plots across the study area [12]. All sampling plots were placed in the center of each forest fragments to minimize edge effects, maintaining a minimum distance of 50 m between plots. We measured the height and diameter at breast height (DBH) of all trees with DBH ≥ 5 cm using a clinometer and measuring tape and considered large trees as those with a DBH ≥ 30 cm. We also assessed the vertical stratification of foliage using a visual estimation based on a modified point-intercept method [41]. Vertical profiles were recorded at three randomly selected points within each plot by estimating the total length (in centimeters) occupied by foliage along a vertical line, divided into six forest strata, each representing a 5 m interval up to 30 m in height. For analysis, we used the average foliage length recorded in each stratum. In the present study, we included only the forest structure data from the 20 sites where bats were sampled.

### 2.4. Bat Sampling

Phyllostomidae bats were sampled through 20 previously selected sites between March and November 2018. The Phyllostomidae bats were captured using mist nets (Avinet research supplies, USA), a more effective methodology for capturing leaf-nosed bats (Phyllostomid Family) [33,42]. At each site, a transact of 120 m was allocated at least 40 m from the edge of the fragment. The sites were sampled in two sessions comprising two days per session. Ten mist nets (12 m long × 2.5 m length) were used on each sample site, following the protocol to increase the possibility of capturing more species [43]. Mist nets were open at dusk, 17 h, and closed at 22 h, being exposed for five hours every night and checked at intervals of 15–30 min. The captured specimens were removed from the mist nets and put into packaged cotton bags to identify each one. The species identification followed the criteria of Vizotto and Taddei (1973), Simmons and Voss (1998), and Gardner (2008) [44,45,46], while the nomenclature and taxonomy followed Simmons (2005), Gardner (2008), and Nogueira et al. (2014) [46,47,48]. All procedures described above were authorized by license issued by the Chico Mendes Institute for Biodiversity Conservation—ICMBio—through the Sisbio licence No. 53792-1.

### 2.5. Data Analysis

We used richness and abundance as components of phyllostomid bat diversity in the analyses. For the sampling sites, we tested spatial autocorrelation between the studied fragments using the Mantel Test [49]. To analyze the forest structure, we performed a principal component analysis (PCA), using the values of the first axis for the analyses. The PCA was based on the four forest structure variables listed earlier (tree height, DBH, large tree density, and foliage stratification) (Table S1). For forest cover, we used the percentage amount of forest in the landscape cover by 800 m radius, following the previous studies [12] around the sampling site, and the same method was used to calculate the amount of pasture in the landscape.

We used structural equation modeling (SEM) to investigate the direct and indirect factors influencing local phyllostomid bat diversity. SEM is a flexible method that facilitates causal understanding by incorporating multiple independent and dependent variables. It was employed to assess the effects of forest cover on components of community structure. We ran SEM analysis with 10,000 bootstrap resamples and assessed model fit using χ^2^, RMSEA, and CFI indices. To identify the best-fitting models, we applied model selection using the Akaike information criterion (AIC) and its corrected version (AICc), with the best model being the one with the lowest ΔAIC value. The GLMs were run using the ‘bbmle’ package [50], while the ‘segmented’ package [51] was used for model selection. All analyses (Appendix A) were performed in the R Studio software version 4.4 [52], using vegan packages [53], nlme [54], plspl [55], and devtools [56].

## 3. Results

The study area comprised 6000 m^2^.h where 596 phyllostomid bats were captured, belonging to six subfamilies, 15 genera and 20 species. The Phyllostominae and Stenodermatinae subfamilies had the highest species richness, with seven and six species, respectively. The frugivorous group was the most captured, with 570 captures, and presented a higher richness, with 10 species. The most abundant species were *Carollia perspicillata* followed by *Rhinophylla pumillio* and *Artibeus obscurus*, with 268 and 142 captures, and 57 captures, respectively. These three species of bats, together, accounted for 78.36% of the total catch (Table 1).

**Figure 2:**
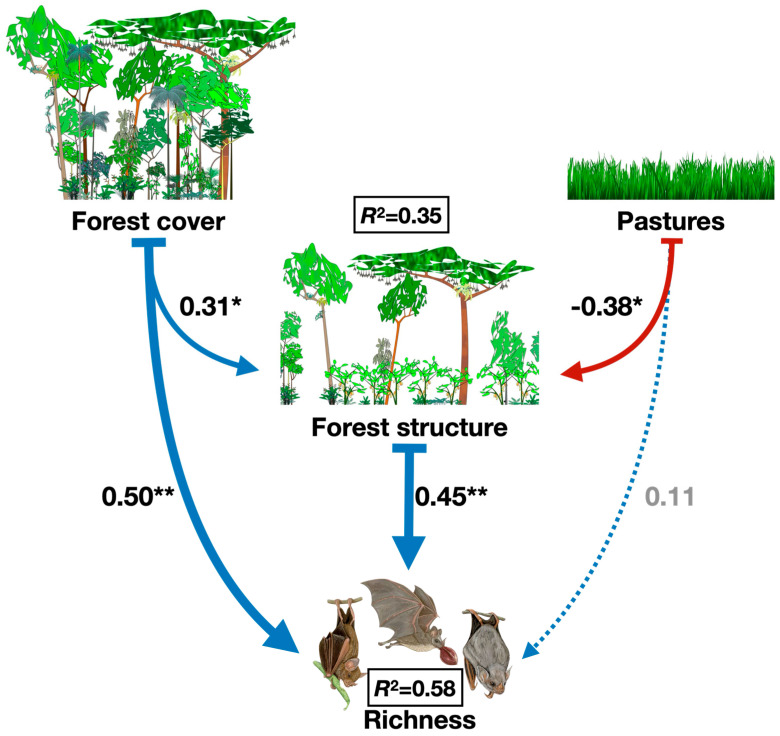
Best-fitted path model showing the direct and indirect effects of percentage of forest cover, percentage of pasture cover, and local forest structure (first PCA axis) on the richness of Phyllostomid bats collected in 20 forest remnants located in southern Bahia, Brazil. Arrow thickness exhibits the relative strength of effects, dashed lines exhibit no significant effect, and *p*-values of coefficients are indicated with asterisks (* *p* < 0.10, ** *p* < 0.05). The black squares show the coefficient of determination (R^2^) for richness of Phyllostomid bats.

On species richness, forest cover has a significant direct positive effect (β = 0.50, *p* = 0.03), as does forest structure (β = 0.45, *p* = 0.03), and this implies that areas with higher forest cover directly support greater phyllostomid bat species richness. Nonetheless, forest cover also has a positive indirect effect on richness through forest structure (β = 0.31, *p* = 0.15), as forest cover contributes to improved forest structure, which enhances species richness. On the other hand, pasture area negatively affects forest structure (β = −0.38, *p* = 0.08). The direct effect of pasture on richness is positive but nonsignificant (β = 0.11, *p* = 0.57) (Table S2). The SEM explains 35% of the variance in forest structure (R^2^ = 0.35) and 58% of the variance in species richness (R^2^ = 0.58) and this indicates that the model provides a good explanation of the factors affecting richness and forest structure (Figure 2).

**Figure 3:**
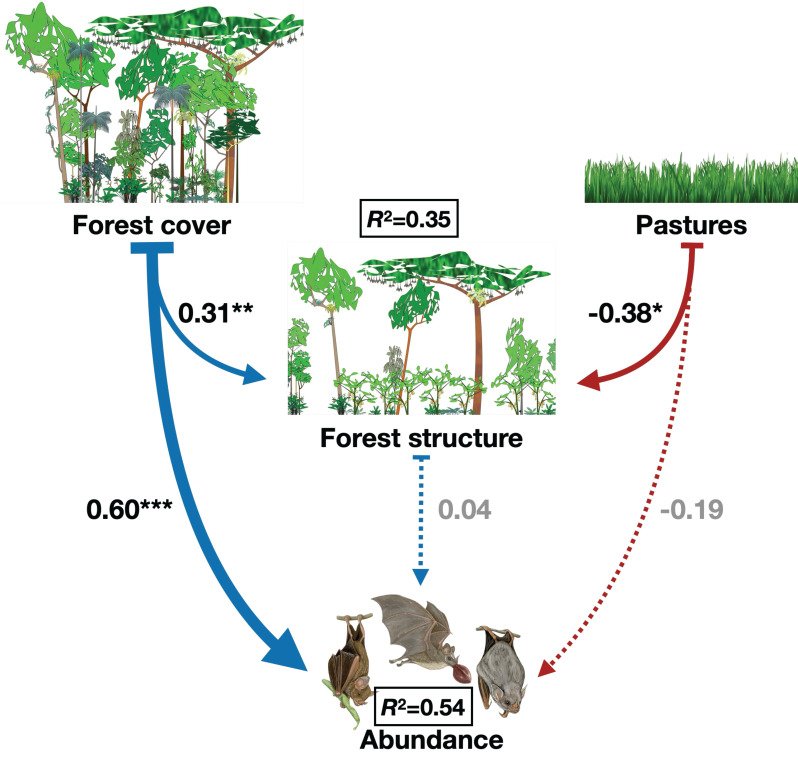
Best-fitted path model showing the direct and indirect effects of percentage of forest cover, percentage of pasture cover, and local forest structure (first PCA axis) on the Phyllostomid bats abundance collected in 20 forest remnants located in southern Bahia, Brazil. Arrow thickness exhibits the relative strength of effects, dashed lines exhibit no significant effect, and *p*-values of coefficients are indicated with asterisks (* *p* < 0.10, ** *p* < 0.05, *** *p* < 0.01). The black squares show the coefficient of determination (R^2^) for abundance of Phyllostomid bats.

On species abundance, forest cover has a strong significant direct positive effect (β = 0.60, *p* < 0.01); however, forest structure has a nonsignificant direct effect on bat abundance (β = 0.04, *p* = 0.82). This highlights that greater forest cover is directly associated with increased bat abundance. On the other hand, forest cover also has a positive effect on forest structure (β = 0.31, *p* = 0.08), but forest structure does not mediate the relationship between forest cover and abundance. Pasture area negatively affects forest structure (β = −0.38, *p* = 0.15), suggesting that increasing pasture areas reduces forest structure. Also, pasture has a negative but nonsignificant direct effect on bat abundance (β = −0.19, *p* = 0.53), indicating a weak negative impact of pasture on abundance that is not statistically robust (Table S3). The SEM explains 35% of the variance in forest structure (R^2^ = 0.35) and 54% of the variance in bat abundance (R^2^ = 0.54) and these values suggest the model provides a moderate-to-good explanation of the factors driving forest structure and phyllostomid bat abundance (Figure 3).

## 4. Discussion

Our findings demonstrate that forest cover plays a central role in maintaining phyllostomid bat diversity in cocoa agroforestry landscapes. Structural equation modeling (SEM) revealed that forest cover directly enhances both species richness and abundance, and it also indirectly contributes to bat diversity by promoting greater forest structural complexity. Although the indirect effect was weaker, it highlights the interconnectedness of landscape and habitat features. In contrast, pasture had a negative impact on forest structure and showed no significant direct or indirect influence on bat diversity, suggesting limited ecological value for these species.

These results underscore the importance of conserving forest cover not only for its direct contribution to bat communities but also for its role in sustaining habitat quality. Forested landscapes tend to retain key vegetation layers and microhabitats essential for forest-dependent bats [32]. In cocoa agroforestry systems, higher surrounding forest cover helps mitigate edge effects and enhances habitat connectivity, supporting the complex vertical and horizontal structure that phyllostomid bats rely on for roosting, foraging, and movement.

The direct and positive effect of forest cover on bat diversity (richness and abundance) aligns with earlier studies highlighting the role of forested habitats in maintaining bat populations in tropical landscapes [10,11]. Forests provide essential resources such as fruit, nectar, and insect prey, as well as roosting sites in hollow trees and dense vegetation. Cocoa agroforestry systems, which retain forest-like characteristics, serve as biodiversity-friendly landscapes, supporting species that are sensitive to habitat loss and fragmentation [9]. Thus, strategies to preserve forest cover in agroforestry-dominated regions are key to sustaining bat diversity.

We also observed a negligible direct effect of forest structure on bat diversity, contrary to our prediction. While structural complexity is often associated with species richness and ecological interactions, our results suggest that forest structure may act more as a mediating variable influenced by forest cover rather than a direct driver of bat diversity faria [10]. This may be due to the dominance of generalist phyllostomid species (such as *Carollia perspicillata* following by *Rhinophylla pumillio* and *Artibeus obscurus*) in our study area, which can adapt to varying levels of structural complexity, as seen in other tropical regions [33]. Future studies should further investigate the role of specific forest structural elements, such as canopy cover and understory density, in shaping bat community dynamics.

Pasture, as predicted, negatively influenced forest structure, highlighting the detrimental impact of land-use intensification on local habitat features [34]. Pastures simplify the landscape, reducing vegetation complexity and connectivity, which, in turn, disrupts the ecological integrity of adjacent forest fragments. However, the direct and indirect effects of pasture on bat diversity were weaker than expected. This finding might reflect the resilience of generalist bat species to pasture-dominated landscapes or the mitigating influence of nearby forest patches. Nevertheless, reducing pasture expansion and promoting reforestation in degraded areas remain essential to conserving forest structure and biodiversity.

Our results further underscore the indirect role of forest cover in supporting bat diversity by maintaining forest structure, as predicted [10,11]. By promoting vegetation layers and connectivity, forest cover enhances habitat quality and foraging opportunities for bats. This indirect pathway emphasizes the interconnected nature of landscape and local factors in shaping biodiversity patterns. In contrast, the indirect negative effect of pasture on bat diversity through forest structure was weaker than anticipated, suggesting that landscape-level conservation strategies may have buffered these impacts.

We acknowledge several limitations in our study that may influence the interpretation of our findings. First, the sample size was relatively limited, which may reduce the generalizability of the results across broader spatial and temporal scales. Although we identified bats at the species level, morphological measurements (e.g., forearm length, body mass) were not recorded, and we were unable to assess ecological responses among guilds, particularly for rare species due to their low capture rates. Furthermore, abiotic factors such as rainfall, moon phase, and seasonality (e.g., dry vs. wet season) were not included in the analysis, which could have influenced bat activity and capture success. Future research incorporating a larger number of sampling sites, environmental covariates, and temporal replication would help refine the understanding of how landscape and local variables shape bat assemblages.

## 5. Conclusions

Here, we highlight the critical role of forest cover in preserving phyllostomid bat diversity in Atlantic Forest remnants in southern Bahia. Sustainable land-use practices that maintain forest patches, reduce pasture expansion, and integrate forest restoration into agricultural mosaics can mitigate the negative impacts of deforestation and fragmentation. Given the ecological importance of phyllostomid bats as pollinators and seed dispersers, conserving their habitats is not only vital for biodiversity but also for ecosystem resilience and sustainability in this biodiversity hotspot.

## Figures and Tables

**Table 1:** Richness, composition, and abundance of Phillostomidae bats captured in the Atlantic Forest landscapes of Southern Bahia, Brazil. Trophic guilds: c = carnivore; H = hematophagous; N = Nectarivore; IC = insectivorous picker; O = omnivorous; F = frugivorous. Number of captures (N) and percentage of capture (%).

Species	Trophic Guilds	N	(%)
**Family Phyllostomidae**			
**Subfamily Desmodontinae**			
*Desmodus rotundus* (É. Geoffroy, 1810)	H	4	0.67
**Subfamily Glossophaginae**			
*Glossophaga soricina* (Pallas, 1766)	N	7	1.17
*Lonchophyla* sp.	N	1	0.17
**Subfamily Phyllostominae**			
*Chrotopterus auritus* (Peters, 1856)	C	3	0.50
*Micronycteris megalotis* (Gray, 1842)	Ic	1	0.17
*Micronycteris* sp.	Ic	1	0.17
*Phylloderma stenops* (Peters 1865)	O	3	0.50
*Phyllostomus discolor* (Wagner, 1843)	O	3	0.50
*Gardenericterys crenulatum* (É. Geoffroy, 1803)	Ic	1	0.17
*Lophostoma brasiliensis* (Peters, 1866)	Ic	2	0.34
**Subfamily Carolliinae**			
*Carollia brevicauda* (Linnaeus, 1758)	F	12	2.01
*Carollia perspicillata* (Linnaeus, 1758)	F	268	44.97
**Subfamily Rhinophyllinae**			
*Rhinophylla fischerae* (Carter, 1966)	F	3	0.50
*Rhinophylla pumilio* (Peters, 1865)	F	142	23.83
**Subfamily Stenodermatinae**			
*Artibeus lituratus* (Olfers, 1818)	F	33	5.54
*Artibeus planirostris* (Spix, 1823)	F	10	1.68
*Artibeus obscurus* (Schinz, 1821)	F	57	9.56
*Dermanura cinerea* (Gervais, 1856)	F	40	6.71
*Platyrrhinus lineatus* (É. Geoffroy, 1810)	F	1	0.17
*Sturnira lilium* (É. Geoffroy, 1810)	F	4	0.67
**TOTAL:**		**596**	**100**

## Data Availability

Data will be made available on request.

## Supplementary Materials

The following supporting information can be downloaded at https://www.mdpi.com/article/10.3390/ani15142082/s1. Table S1: Forest structure variables used in the PCA: (1) mean tree height, (2) DBH of trees ≥ 5 cm, (3) density of large trees (DBH ≥ 30 cm), and (4) average foliage coverage per forest stratum (from vertical stratification estimates); Table S2: Structural Equation Model Results for Richness. Summary of path coefficients and model estimates for the SEM predicting forest structure and species richness.; Table S3: Structural Equation Model Results for abundance. Summary of path coefficients and model estimates for the SEM predicting (a) forest structure and (b) bat abundance.

## Appendix A

### Appendix A.1

The script models used structural equation modeling (SEM) to investigate the direct and indirect effects of landscape factors (forest cover and pasture) and local factors (forest structure) on the diversity (richness and abundance) of phyllostomid bats across 20 forest fragments in the Atlantic Forest of southern Bahia, Brazil.

The **Script Models**

**Richness**

dados=read.table(“R_auberico.csv”,header=T,sep=“;”,dec=“,”)

head(dados)

install.packages(“vegan”)

install.packages(“plspm”)

library(vegan)

install.packages(“devtools”)

library(devtools)

install_github(“gastonstat/plspm”, force=T)

library(plspm)

path.riq=cbind(log(dados$R_800+1), log(dados$Pasture+1), dados$PC1.estr.vert,dados$species)

colnames(path.riq)=c(“Forest_cover”, “Pasture”,”Forest_structure”,”Richness”)

path.riq=decostand(path.riq, “standardize”)

pairs(path.riq)

forest_cover=c(0,0,0,0)

pasture=c(0,0,0,0)

forest_structure=c(1,1,0,0)

richness=c(1,1,1,0)

rich_path=rbind(forest_cover,pasture,forest_structure,richness)

colnames(rich_path)=rownames(rich_path)

rich_path

rich_blocks = list(1,2,3,4)

rich_modes=rep(“B”,4)

rich_pls = plspm(path.riq, rich_path, rich_blocks, modes =rich_modes,boot.val=T,br=10000,scaled=T)

rich_pls

summary(rich_pls)$boot$paths #individual coefficients

summary(rich_pls)$boot$rsq

summary(rich_pls) #individual coefficients

rich_pls$path_coefs

rich_pls$inner_model

paths.resize.rich = rich_pls$path_coefs

arrow_lwd = 10 * abs(round(paths.resize.rich, 2))

plot(rich_pls, arr.pos = 0.35, arr.lwd = arrow_lwd)

dev.off()

########

* *

**Script Models**

**Abundance**

* *

dados=read.table(“R_auberico.csv”,header=T,sep=“;”,dec=“,”)

head(dados)

library(vegan)

library(plspm)

path.abu=cbind(log(dados$R_800+1), log(dados$Pasture+1), dados$PC1.estr.vert,log(dados$individuals+1))

path.abu=decostand(path.abu1,”standardize”)

colnames(path.abu)=c(“Forest_cover”, “Pasture”,”Forest_structure”,”Abundance”)

pairs(path.abu)

colnames(path.abu)=c(“Forest_cover”, “Pasture”,”Forest_structure”,”Richness”)

pairs(path.abu)#de um alt+tab aqui pra ver a janela externa

forest_cover=c(0,0,0,0)

pasture=c(0,0,0,0)

forest_structure=c(1,1,0,0)

abundance=c(1,1,1,0)

abund_path=rbind(forest_cover,pasture,forest_structure,abundance)

colnames(abund_path)=rownames(abund_path)

abund_path

innerplot(abund_path)

dev.off()

abund_blocks = list(1,2,3,4)

abund_modes=rep(“B”,4)

abund_pls = plspm(path.abu, abund_path, abund_blocks, modes =abund_modes,boot.val=T,br=10000,scaled=T)

abund_pls

summary(abund_pls)$boot$paths#individual coefficients

summary(abund_pls)$boot$rsq

summary(abund_pls) #individual coefficients

abund_pls$path_coefs

abund_pls$inner_model

paths.resize.abund = abund_pls$path_coefs

arrow_lwd = 10 * abs(round(paths.resize.abund, 2))

plot(abund_pls, arr.pos = 0.35, arr.lwd = arrow_lwd)

dev.off()

#######
