# Supplementary material for: Landscape Composition and Forest Structure Shape Phyllostomid Bat Assemblages in the Atlantic Forest Remnants
Source: Animals (Basel). 2025 Jul 15;15(14):2082. doi: 10.3390/ani15142082 (PMC12291667; doi:10.3390/ani15142082)
Supplement: Supplementary file 1 [file animals-15-02082-s001.zip › animals-3724601-supplementary.pdf]

Table S1. Forest structure variables used in the PCA: (1) mean tree height, (2) DBH of trees ≥ 5 cm, (3) density of large trees (DBH ≥ 30 cm), and (4) average foliage coverage per forest stratum (from vertical stratification estimates).

Sites	Average foliage coverage per forest stratum						DBH of trees ≥ 5 cm	Mean tree height	Density of large trees (DBH ≥ 30 cm)
	0_5 m	5_10 m	10_15m	15_20 m	20_25 m	25_30 m			
Site 1	337	248	183	83	0	0	12,4	12,9	93,8
Site 2	373	264	215	91	0	0	9,8	10,2	0
Site 3	463	320	135	0	0	25	10,8	9,1	22,2
Site 4	234	224	222	50	0	0	11,5	9,5	58,3
Site 5	387	317	184	110	8	0	11,7	9,8	94,4
Site 6	343	283	241	50	0	0	13	9,2	106,3
Site 7	391	310	232	83	15	0	13,4	9,6	111,1
Site 8	257	284	312	70	10	0	10,8	9,7	86,1
Site 9	150	135	250	250	0	0	12,3	10,2	155,6
Site 10	176	226	337	248	34	30	13,1	10,4	147,2
Site 11	165	251	307	361	60	0	15,6	10,7	225
Site 12	355	345	205	0	0	0	10,9	7,8	44,4
Site 13	279	198	85	50	0	125	11,7	8,7	91,7
Site 14	102	100	210	367	145	50	13,4	11,8	188,9
Site 15	229	435	298	100	7	0	12,3	10,1	158,3
Site 16	168	201	256	330	90	0	11,2	10,2	31,3
Site 17	138	184	261	198	92	16	10,7	10	116,7
Site 18	352	272	261	267	12	0	12	10,7	218,8
Site 19	170	208	251	73	15	0	12,4	11	138,9
Site 20	337	278	186	173	142	191	12,8	13	220

Table S2. Structural Equation Model Results for Richness. Summary of path coefficients and model estimates for the SEM predicting forest structure and species richness.

Path Coefficients

	Forest Cover	Pasture	Forest Structure	From: Richness
Forest Structure	0.318	-0.387	0	0
Richness	0.502	0.112	0.449	0

Model Estimates

(a) Response Variable: Forest Structure

Predictor	Estimate	Std. Error	t value	Pr(>|t|)
Intercept	-3.27×10^{-17}	0.1947	-1.68×10^{-16}	1.0000
Forest cover	0.318	0.2151	1.48	0.1580
Pasture	-0.387	0.2151	-1.80	0.0897

(b) Response Variable: Richness

Predictor	Estimate	Std. Error	t value	Pr(>|t|)
Intercept	-1.37×10^{-17}	0.1613	-8.51×10^{-17}	1.0000
Forest cover	0.502	0.1893	2.65	0.0174
Pasture	0.112	0.1944	0.576	0.5726
Forest structure	0.449	0.2009	2.24	0.0399

Table S3. Structural Equation Model Results for abundance. Summary of path coefficients and model estimates for the SEM predicting (a) forest structure and (b) bat abundance.

Path Coefficients

	Forest cover	Pasture	Forest Structure	Abudance
Forest cover	0	0	0	0
Pasture	0	0	0	0
Forest Structure	0.317720	-0.3871558	0	0
Abudance	0.603283	-0.1985199	0.04741005	0

(b) Model: Forest Structure

Predictor	Estimate	Std. Error	t value	Pr(>|t|)
Intercept	-3.37×10^{-17}	0.1947	-1.73×10^{-16}	1.0000
Forest cover	0.318	0.2151	1.48	0.0897
Pasture	-0.387	0.2151	-1.80	0.1580

(c) Model: Bat Abundance

Predictor	Estimate	Std. Error	t value	Pr(>|t|)
Intercept	-2.12×10^{-16}	0.1687	-1.26×10^{-15}	1.0000
Forest cover	0.603	0.1979	3.05	0.0077
Pasture	-0.199	0.2033	-0.98	0.5334
Forest structure	0.474	0.2101	2.26	0.8243
